# The Impact of Time Interval to Surgery After Neoadjuvant Chemoradiotherapy on Oncological Outcomes: A Long‐Term Follow‐Up Study on Rectal Cancer Patients

**DOI:** 10.1002/cnr2.70493

**Published:** 2026-02-17

**Authors:** Marzieh Rahimi, Mohammadreza Mamaghani‐Ghazijahani, Fatemeh Shahabi, Majid Ansari, Mahdie Ghiyasi Noei, Mina Alvandipour, Abbas Abdollahi

**Affiliations:** ^1^ Endoscopic and Minimally Invasive Surgery Research Center Mashhad University of Medical Sciences Mashhad Iran; ^2^ Department of Surgery, Imam Khomeini Hospital Mazandaran University of Medical Sciences Sari Iran

**Keywords:** interval, neoadjuvant chemoradiotherapy, rectal cancer, recurrence, surgery, survival

## Abstract

**Background:**

Neoadjuvant chemoradiotherapy (nCRT) has been shown to improve prognosis in patients with locally advanced rectal cancer (LARC). However, there is still debate regarding the optimal time of surgery following nCRT.

**Aims:**

This exploratory study aimed to investigate whether there is a difference in oncological outcomes between patients with LARC who undergo surgery within 6 weeks of nCRT and those who wait longer than 6 weeks.

**Methods and Results:**

This retrospective study included patients with rectal tumors who underwent nCRT followed by laparoscopic surgery during 2011–2020. Based on the time interval, the study participants were divided into two groups: ≤ 6 weeks and > 6 weeks. Receiver Operating Characteristics and Kaplan–Meier survival curves were employed to evaluate the effect of time interval on overall survival (OS) and recurrence‐free survival (RFS). Cox regression analysis was used to identify the prognostic factors of RFS and OS. A total of 175 patients were included in our study with a mean ± SD age of 56.1 ± 12.9 years. The median (interquartile range) follow‐up time was 71 (63) months. There was no difference in demographic and clinical variables between the two groups. Neither pCR, OS, nor RFS was affected by the nCRT‐surgery interval. Multivariable Cox regression analysis showed that patients with ypStage III had a mortality and recurrence hazard of 7.6 and 3.3 times higher than those with pCR (*p* < 0.001, *p* = 0.018, respectively). Additionally, those who underwent abdominoperineal resection (APR) were 5.8 times more at risk of developing recurrence (*p* = 0.001).

**Conclusion:**

The present study revealed that pCR rates, OS, and RFS were not affected by the nCRT‐surgery interval within the cohort studied. In addition, the time interval of more than 6 weeks may be safe, as no substantial difference in postoperative complications was observed between groups. The ypTNM staging was an independent predictor of OS and RFS. Moreover, APR was considered an important prognostic factor for RFS. These findings require further investigation by multicenter, large‐scale studies in the future.

## 1 Introduction

Rectal cancer represents approximately 30% of all colorectal malignancies. More than 70% of the rectal cancer patients exhibit locally advanced disease, which diminishes the likelihood of cure [1, 2]. The standard management of locally advanced rectal cancer (LARC) comprises a combination of neoadjuvant therapy and surgery [3]. Neoadjuvant therapy is recommended in patients with cancer stages cT3–T4, tumors with nodal involvement, extramural venous invasion, and threatened circumferential resection margin (CRM) [4]. The usefulness of neoadjuvant chemoradiotherapy (nCRT) was demonstrated by the German CAO/ARO/AIO 94 randomized controlled trial (RCT) [5]. Neoadjuvant CRT is used to shrink the tumors, enhance the odds of R0 resection or sphincter‐preserving procedure (SPP), and reduce the risk of local recurrence in LARC [6]. In addition, nCRT may completely eradicate all tumor cells from the rectal wall and mesorectum, representing a pathologic complete response (pCR). The pCR is currently considered a surrogate indicator of recurrence‐free survival (RFS) in LARC. Furthermore, this subgroup may benefit from the ‘watch‐and‐wait’ strategy instead of undergoing radical surgery [7, 8, 9].

With the growing use of neoadjuvant therapies, it becomes more important to determine the optimal nCRT‐surgery interval to achieve a long‐lasting disease‐free survival for the majority of patients [7]. However, the optimal waiting period is still up for debate. The first prospective RCT on this matter was conducted as the Lyon R90‐01 trial [10]. They found that the long interval group (6–8 weeks) showed significantly better pathologic downstaging and clinical tumor response compared to the short interval group (2 weeks), while no significant differences were observed regarding postoperative complications, SPPs, short‐term survival, or local recurrence between groups. Then, 6–8 weeks of interval from nCRT to surgery was widely accepted in clinical practice. Consensus‐based guidelines including the National Comprehensive Cancer Network suggest 5–12 weeks and the European Society of Medical Oncology recommends 4–12 weeks for the waiting period [11, 12]. A majority of surgeons would like to perform surgery about 6 weeks following the end of nCRT [1]. The rationale of postponing surgery derives from the principles of radiotherapy; DNA damage happens during irradiation, while cellular lysis occurs in subsequent weeks [13]. Current studies on nCRT‐surgery interval, including RCTs, mostly focus on improving the pCR rates. And there has been less focus on determining the optimal time interval to improve oncological prognosis. The present study was designed to investigate the effect of nCRT‐surgery interval on oncological outcomes in LARC patients in a long‐term follow‐up.

## 2 Materials and Methods

### 2.1 Patients and Study Setting

In this retrospective cohort analysis, we included LARC patients who underwent nCRT followed by surgery at Razavi Hospital, Mashhad, Iran, from July 1, 2011, to December 10, 2020. The study participants underwent three different surgical procedures: abdominoperineal resection (APR), transabdominal specimen extraction (TASE), and natural orifices specimen extraction (NOSE). Patients with upper or middle rectal cancer commonly underwent TASE surgery, whereas those with low rectal tumors underwent NOSE or APR surgery. NOSE procedure was generally performed for individuals who were eligible for sphincter preservation, while APR was conducted in other patients. In the laparoscopic approach with the modified lithotomy position, after complete exploration of the abdomen, the inferior mesenteric artery was exposed and then high‐ligated. The inferior mesenteric vein was subsequently ligated at the inferior border of the pancreas, adjacent to the duodenum. The splenic flexure, descending colon, sigmoid, and rectum were all fully mobilized. In the NOSE technique, a circular incision was made just above the dentate line and far enough distal to the inferior margin of the rectal mass. Following complete dissection, a wound protector was placed, and the rectum, sigmoid, and descending colon were pulled through the anus. Resection was performed with an adequate proximal margin, and the rectal mass was excised. An anastomosis was made between the descending colon and the distal rectum/anal canal using either stapler or hand‐sewn techniques. In the TASE technique, following complete mobilization, the rectum was transected with a stapler at an adequate distal margin. The tumor was then removed through a wound protector with a Pfannenstiel incision from the abdomen. In the APR technique, a colostomy was created in the left lower quadrant of the abdomen following tumor excision. Study participants were followed up based on established surveillance protocols of rectal cancer for the 5 years following surgery. Since then, annual phone call follow‐ups have been conducted. The last date of follow‐up was in 2023.

### 2.2 Inclusion and Exclusion Criteria

The study participants were required to fulfill the subsequent criteria: (1) All patients were diagnosed with biopsy‐confirmed rectal cancer located within 15 cm from the anal verge using colonoscopy; (2) Tumors were assessed using thoracic and abdominopelvic enhanced computed tomography (CT) scan and categorized as non‐metastatic (stage cM0) at the time of initial evaluation; (3) The patients underwent laparoscopic curative surgery after completing nCRT; and (4) The exact date of nCRT completion and surgery was available. The exclusion criteria were the interval from nCRT to surgery was less than 1 week or more than 20 weeks.

### 2.3 Neoadjuvant Treatment

The nCRT regimen of our patients comprised Capecitabine 1 g/m2 twice daily, along with 28 sessions of radiotherapy, with a cumulative radiation dose of 50.4 Gray (Gy).

### 2.4 Variables

Demographic and clinical characteristics including age, gender, tumor location (categorized as lower, middle, and upper rectum based on distance from the anal verge: 0 to 5 cm, 6 to 10 cm, 11 to 15 cm, respectively), surgical techniques, CRM status, distal resection margins (DRM) status, ypTNM staging, pCR achievement, and postoperative complications including obstruction, pelvic abscess/collection, and anastomosis failure were reported. In addition, adjuvant therapy status, recurrence status (Yes/No), recurrence pattern (locoregional recurrence (LR), distant metastasis (DM), and DM plus LR), follow‐up time, and mortality were reported in this study. Since all the study participants had rectal cancer, pelvic recurrence was regarded as LR. A combination of methods was used for recurrence diagnosis, including physical examinations, checking tumor marker levels, imaging modalities (like CT or positron emission tomography scans), and confirming through biopsy.

### 2.5 Outcomes

Primary endpoints of our study were the pCR achievements, overall survival (OS), and RFS, known as oncologic outcomes, and secondary endpoints were surgical outcomes. In our study, pCR was defined as the absence of any residual cancer cells in the resected specimen, specifically characterized by a tumor regression score of 0 or a classification of ypT0N0. The duration from the surgery date to either the date of death or the last follow‐up was regarded as OS, and the time from the surgery date to the date of recurrence or the last follow‐up was considered RFS.

### 2.6 Statistical Analysis

Categorical variables were reported as frequency (percentage). Quantitative variables with normal distribution were expressed as mean ± standard deviation (SD), and non‐normal variables were reported as median (interquartile range, IQR). To compare categorical variables, after checking relevant assumptions, the *χ* ^2^ test or Fisher's exact test was used. After checking the normality, independent sample *t*‐test or Mann–Whitney *U* test was used to analyze continuous variables, as applicable. The classification role of nCRT‐surgery interval in death and recurrence outcomes was evaluated by Receiver Operating Characteristics (ROC) curve analysis. The Kaplan–Meier survival curve was used to illustrate OS and RFS times and compared between two interval time groups. Log‐rank test was employed to examine the statistical differences between groups in survival curves. We used Cox regression analysis to evaluate the effect of variables on RFS and OS. SPSS version 26.0 (Chicago, IL, USA) was employed for statistical analysis. The significance level was considered 0.05.

## 3 Results

**FIGURE 1:**
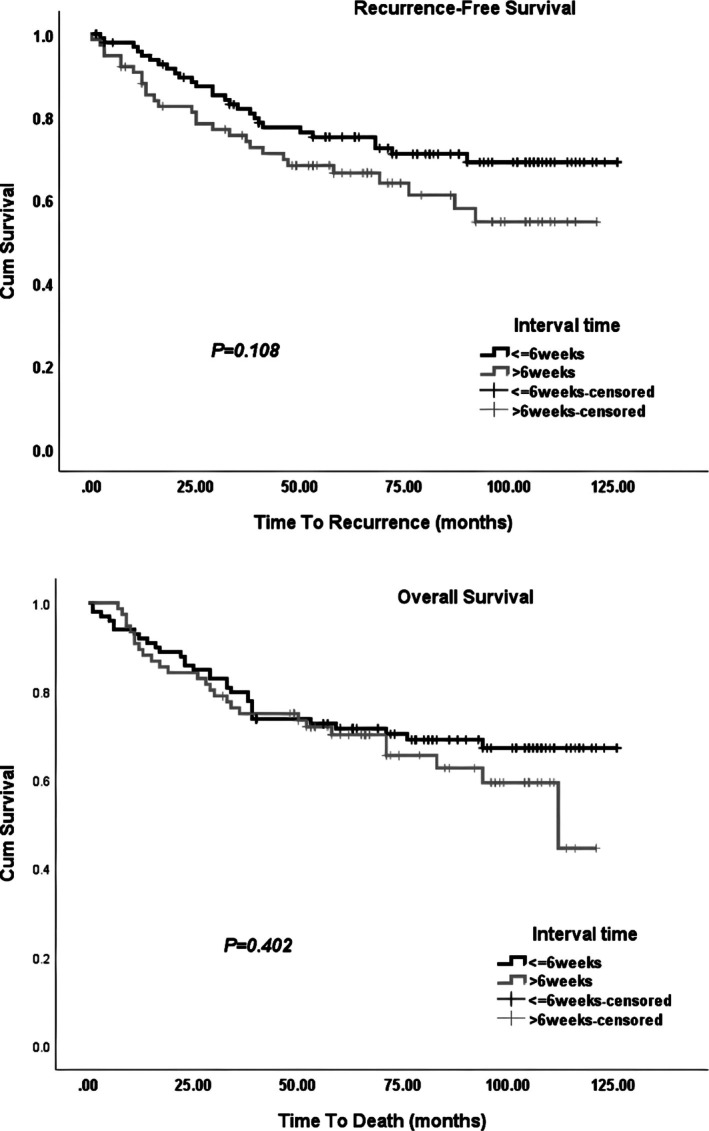
Kaplan–Meier curves of recurrence‐free survival and overall survival in the ≤ 6‐week group and > 6‐week group with long‐term follow‐up.

In this study, 175 eligible patients were enrolled with a mean ± SD age of 56.1 ± 12.9 years. The median (IQR) nCRT‐surgery interval was 6 (4) weeks. Of the patients, 30 (17.1%) had APR, 97 (55.4%) had NOSE, and 48 (27.4%) had TASE surgery. There was no conversion to open surgery in our study. In the TASE and NOSE groups, 96 (66.2%) patients underwent protective ostomy. Demographic and clinical characteristics of all patients were compared between two determined groups based on the median waiting period (Table 1). There were no significant differences between groups in terms of demographic and clinical variables (all *p*‐values > 0.05). The comparison of clinical outcomes based on time interval is shown in Table 2. Recurrence rates (*p* = 0.176), pCR achievement rates (*p* = 0.926), and postoperative complications (*p* > 0.05) were independent of nCRT‐surgery interval. The median (IQR) follow‐up duration was 71 (63) months. It was significantly higher in patients undergoing surgery ≤ 6 weeks following nCRT (*p* = 0.034). RFS and OS were compared between defined groups using log‐rank test and visualized by Kaplan–Meier curves (Figure 1). Neither OS (*p* = 0.402) nor RFS (*p* = 0.108) was influenced by nCRT‐surgery interval.

**TABLE 1:** The comparison of baseline demographic and clinical characteristics between two groups of patients based on time interval from neoadjuvant chemoradiotherapy to surgery.

Characteristics		Overall (*N* = 175)	≤ 6 weeks (*N* = 99)	> 6 weeks (*N* = 76)	*p*
Age at diagnosis, mean ± SD, years		56.1 ± 12.9	55.5 ± 12.4	57.1 ± 13.6	0.438
Gender, *N* (%)	Female	66 (37.7)	38 (38.4)	28 (36.8)	0.835
	Male	109 (62.3)	61 (61.6)	48 (63.2)	
Tumor location, *N* (%)	Lower	100 (57.1)	55 (55.6)	45 (59.2)	0.877
	Middle	54 (30.9)	32 (32.3)	22 (28.9)	
	Upper	21 (12)	12 (12.1)	9 (11.8)	
Surgical techniques resection, *N* (%)	NOSE	97 (55.4)	57 (57.6)	40 (52.6)	0.270
	TASE	48 (27.4)	29 (29.3)	19 (25)	
	APR	30 (17.1)	13 (13.1)	17 (22.4)	
ypTNM staging^a^, *N* (%)	pCR (Stage 0)	50 (28.6)	28 (28.3)	22 (28.9)	0.934
	ypT1–2 N0 (Stage I)	36 (20.6)	22 (22.2)	14 (18.4)	
	ypT3–4 N0 (Stage II)	34 (19.4)	19 (19.2)	15 (19.7)	
	ypT0–4 N+ (Stage III)	48 (27.4)	26 (26.3)	22 (28.9)	
Distal resection margin involvement^a^, *N* (%)	Free	163 (93.1)	91 (91.9)	72 (94.7)	0.792
	Close (Distance < 0.5–1 mm)	4 (2.3)	3 (3)	1 (1.3)	
	Involved	1 (0.6)	1 (1)	0	
Circumferential resection margin involvement^a^, *N* (%)	Free	165 (94.3)	94 (94.9)	71 (93.4)	0.720
	Close (Distance < 0.5–1 mm)	1 (0.6)	0	1 (1.3)	
	Involved	2 (1.1)	1 (1)	1 (1.3)	
Adjuvant receipt status, *N* (%)	Yes	141 (80.6)	82 (82.8)	59 (77.6)	0.389
	No	34 (19.4)	17 (17.2)	17 (22.4)	

Abbreviations: APR, abdominoperineal resection; pCR, pathologic complete response; TASE: transabdominal specimen extraction.

^a^ Pathology results were missing for seven patients. Significant at *α* = 0.05. NOSE: natural orifices specimen extraction.

**TABLE 2:** The comparison of clinical outcomes between two groups of patients based on time interval from neoadjuvant chemoradiotherapy to surgery.

Outcomes		Overall (*N* = 175)	≤ 6 weeks (*N* = 99)	> 6 weeks (*N* = 76)	*p*
Follow‐up time, median(IQR), months		71 (63)	80 (66)	59 (59)	**0.034** ^**b**^
Death, *N* (%)	Yes	58 (33.1)	31 (31.3)	27 (35.5)	0.557
	No	117 (66.9)	68 (68.7)	49 (64.5)	
Recurrence status, *N* (%)	Yes	55 (31.4)	27 (27.3)	28 (36.8)	0.176
	No	120 (68.6)	72 (72.7)	48 (63.2)	
Recurrence pattern, *N* (%)	Locoregional (LR)	18 (10.3)	11 (11.1)	7 (9.2)	0.237
	Distant metastasis (DM)	34 (19.4)	14 (14.1)	20 (26.3)	
	LR and DM	3 (1.7)	2 (2)	1 (1.3)	
pCR achievement^a^, *N* (%)	Yes	50 (28.6)	28 (28.3)	22 (28.9)	0.926
	No	118 (67.4)	67 (67.7)	51 (67.1)	
Postoperative obstruction, *N* (%)	Yes	8 (4.6)	6 (6.1)	2 (2.6)	0.469
	No	167 (95.4)	93 (93.9)	74 (97.4)	
Postoperative pelvic abscess/collection, *N* (%)	Yes	24 (13.7)	11 (11.1)	13 (17.1)	0.253
	No	151 (86.3)	88 (88.9)	63 (82.9)	
Postoperative anastomosis failure, *N* (%)	Yes	20 (11.4)	9 (9.1)	11 (14.5)	0.267
	No	155 (88.6)	90 (90.9)	65 (85.5)	

Abbreviation: pCR, pathologic complete response.

^a^ Pathology results were missing for 7 patients.

^b^ Significant at *α* = 0.05.

**FIGURE 2:**
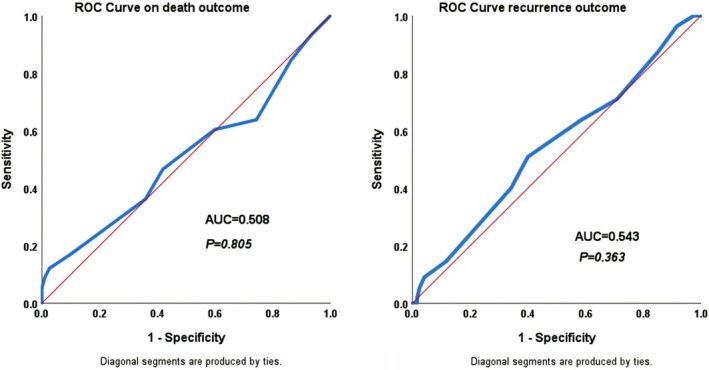
The receiver operating characteristics curve analysis using death and recurrence as outcomes considering time interval from neoadjuvant chemoradiotherapy to surgery (weeks).

To evaluate the factors affecting RFS and OS, univariate Cox regression analysis was employed, considering *α* = 0.2, to identify candidate variables for multivariable analysis (Table 3). Variables, including age, tumor location, surgical technique, ypTNM staging, DRM, and CRM, were considered for inclusion in the multivariable model for both RFS and OS. In addition, Postoperative pelvic abscess/collection and time interval were identified as candidate variables for OS and RFS, respectively. In multivariable Cox regression analysis, patients with ypStage III had a mortality and recurrence hazard ratio (HR) of 7.6 and 3.3 times higher than patients who achieved pCR, in the presence of other variables (*p* < 0.001, *p* = 0.018, respectively) (Table 4). Moreover, those who underwent APR were 5.8 times more at risk of developing recurrence than patients who underwent NOSE surgery (*p* = 0.001). ROC curve analysis revealed that the quantitative variable of nCRT‐surgery interval was neither a significant predictor of mortality (*p* = 0.805) nor recurrence (*p* = 0.363) (Figure 2).

**TABLE 3:** The hazard ratio of recurrence and death using univariate cox regression analysis.

Characteristics		Overall survival		Recurrence free survival	
		Hazard ratio (95% CI)	*p*	Hazard ratio (95% CI)	*p*
Age at diagnosis		1 (1, 1.04)	**0.048**	1 (1, 1.04)	**0.175**
Interval time from chemoradiotherapy to surgery	≤ 6 weeks	—	0.404	—	**0.111**
	> 6 weeks	1.2 (0.7, 2.1)		1.5 (0.9, 2.6)	
Gender	Male	—	0.744	—	0.769
	Female	0.9 (0.5, 1.6)		0.9 (0.5, 1.6)	
Tumor location	Lower	—	—	—	—
	Middle	0.6 (0.3, 1.1)	**0.121**	0.3 (0.1, 0.7)	**0.005** ^**b**^
	Upper	0.8 (0.4, 1.8)	0.639	0.8 (0.3, 1.7)	0.519
Surgical techniques resection	NOSE	—	—	—	—
	TASE	0.6 (0.3, 1.3)	**0.186**	0.6 (0.3, 1.3)	**0.185**
	APR	1.7 (0.9, 3.2)	**0.101**	2.2 (1.1, 4.1)	**0.017** ^**b**^
ypTNM staging	pCR (Stage 0)	—	—	—	—
	ypT1–2 N0 (Stage I)	2.3 (0.8, 6.3)	**0.111**	1.4 (0.5, 3.8)	0.484
	ypT3–4 N0 (Stage II)	2.3 (0.8, 6.3)	**0.122**	1.9 (0.8, 4.9)	**0.160**
	ypT0–4 N+ (Stage III)	7 (2.9, 16.8)	**< 0.001** ^**b**^	5.2 (2.4, 11.6)	**< 0.001** ^**b**^
Distal resection margin involvement^a^	Free	—	**0.227**	—	**0.204**
	Involved/Close (< 0.5–1 mm)	2 (0.6, 6.6)		2.3 (0.7, 6.8)	
Circumferential resection margin involvement^a^	Free	—	**0.141**	—	**0.095**
	Involved/Close (< 0.5–1 mm)	2.9 (0.7, 12)		3.3 (0.8, 13.9)	
Adjuvant receipt status	No	—	0.546	—	0.607
	Yes	1.2 (0.6, 2.4)		1.2 (0.6, 2.4)	
Postoperative obstruction	No	—	0.594	—	0.620
	Yes	0.7 (0.2, 2.8)		0.7 (0.2, 2.9)	
Postoperative pelvic abscess/collection	No	—	**0.133**	—	0.990
	Yes	1.7 (0.8, 3.2)		1 (0.4, 2.2)	
Postoperative anastomosis failure	No	—	0.807	—	0.868
	Yes	0.9 (0.4, 2.1)		0.9 (0.4, 2.2)	

*Note:* Bold formatting: Candidate variables for entering multivariable model.

Abbreviations: APR, abdominoperineal resection; CI, confidence interval; NOSE, natural orifices specimen extraction; pCR, pathologic complete response; TASE, transabdominal specimen extraction.

^a^ Due to the small sample size in the involved and close margin categories, these two categories were merged.

^b^ Significant at *α* = 0.05.

**TABLE 4:** The hazard ratio of recurrence and death using multivariable cox regression analysis.

Characteristics		Overall survival		Recurrence free survival	
		Hazard ratio (95% CI)	*p*	Hazard ratio (95% CI)	*p*
Age at diagnosis		1 (1, 1.05)	0.092	1 (1, 1.01)	0.309
Interval time from chemoradiotherapy to surgery	≤ 6 weeks			—	0.570
	> 6 weeks			0.8 (0.4, 1.6)	
Gender	Male				
	Female				
Tumor location	Lower	—	—	—	—
	Middle	0.8 (0.4, 1.8)	0.640	1.6 (0.6, 4.4)	0.386
	Upper	1 (0.4, 2.4)	0.950	1.5 (0.6, 4.2)	0.399
Surgical techniques resection	NOSE	—	—	—	—
	TASE	0.7 (0.3, 1.4)	0.300	0.8 (0.3, 2)	0.652
	APR	1.4 (0.6, 3.2)	0.422	5.8 (2.1, 15.8)	**0.001** ^**b**^
TNM staging	pCR (Stage 0)	—	—	—	—
	T1,2 (Stage I)	2.1 (0.7, 6)	0.152	1.4 (0.4, 4.2)	0.571
	T3,4 (Stage II)	2.5 (0.9, 7.2)	0.092	2.4 (0.8, 7)	0.107
	N+ (Stage III)	7.6 (3, 18.7)	**< 0.001** ^**b**^	3.3 (1.2, 9)	**0.018** ^**b**^
Distal resection margin involvement^a^	Free	—	0.082	—	0.321
	Involved/Close (< 0.5–1 mm)	3 (0.9, 10.7)		1.9 (0.5, 7.3)	
Circumferential resection margin involvement^a^	Free	—	0.901	—	0.503
	Involved/Close (< 0.5–1 mm)	0.9 (0.2, 4.2)		1.7 (0.4, 8.2)	
Adjuvant receipt status	No				
	Yes				
Postoperative obstruction	No				
	Yes				
Postoperative pelvic abscess/collection	No	—	0.111		
	Yes	1.8 (0.9, 3.8)			
Postoperative anastomosis failure	No				
	Yes				

*Note:* The bold values are those with a < 0.05 (as noted in footnote b).

Abbreviations: APR, abdominoperineal resection; CI, confidence interval; NOSE, natural orifices specimen extraction; pCR, pathologic complete response; TASE, transabdominal specimen extraction.

^a^ Due to the small sample size in the involved and close margin categories, these two categories were merged.

^b^ Significant at *α* = 0.05.

## 4 Discussion

Neoadjuvant CRT has led to higher pCR achievements, reduced risk of local recurrence, as well as an increase in SPPs and improved quality of life due to consistent tumor downstaging [2]. With the growing use of neoadjuvant therapy, determining the optimal nCRT‐surgery interval becomes more important to improve the prognosis of LARC patients. Our retrospective study showed that pCR achievement rates, OS, and RFS were not affected by nCRT‐surgery interval. In addition, the time interval of more than 6 weeks was deemed safe, as no substantial difference in postoperative complications was observed between groups.

Several studies have demonstrated that the extended waiting period was correlated with higher pCR achievement, based on time points of 7 weeks [7], 8 weeks [14], 9 weeks [15], or longer. Nevertheless, there are contradictory results regarding this association [16, 17, 18]. The present study did not find a trend between the extended nCRT‐surgery interval and increased pCR rates. A number of factors may explain this discrepancy. Our pCR rate of 28.6% was greater than the rates reported in prior studies [4, 19, 20]. A large study by Probst et al. [21], including 17 255 patients, reported a pCR rate of 8.7% before 6 weeks and 13.2% after 8 weeks (*p* < 0.001). Despite the improved rate, our result did not support that the pCR rate was affected by the time interval. Prolonged waiting does not alter the inherent characteristics of tumoral lesions or their ability to be eradicated [13]. Gambacorta et al. [22] conducted a pooled analysis of 3085 patients from seven RCTs with a pCR rate of 14% at a median waiting period of 6 weeks. They concluded that 95% of all pCR events were achieved within a minimum of 10 weeks from nCRT. This analysis had the advantage of excluding all non‐randomized data. However, they accrued patients over an extensive period (1993–2014), and there was considerable heterogeneity in chemotherapy agents and radiotherapy dosages that affected the pCR rate. Consequently, the optimal time interval for maximizing the rate, as well as the significance of pCR itself, remains to be established.

A relatively novel strategy for the neoadjuvant treatment of LARC is the integration of systemic chemotherapy either before or after nCRT, referred to as total neoadjuvant therapy (TNT). It promotes tumor downstaging, addresses micrometastasis early on, and prevents distant recurrence, a major cause of mortality in rectal cancer patients [2, 23]. The PRODIGE 23 trial reported a higher pCR rate in patients with LARC receiving TNT than those in the nCRT arm (27.5% vs. 11.7%) [24]. Furthermore, the increasing rates of clinical complete response and pCR with TNT have created prospects for organ preservation and “watch‐and‐wait” strategy in selected patients [25].

In agreement with the previous reports, OS was not influenced by nCRT‐surgery interval in our study. No consistent data exist indicating enhanced OS as a result of prolonged interval [20, 26, 27]. Furthermore, we found no effect of the waiting period on RFS. This could be explained by the similar tumoral response, as shown by ypTNM stage, in both interval groups. A previous meta‐analysis, including 9070 patients from mainly non‐randomized studies, showed that OS, local recurrence, and DM were not affected by nCRT‐surgery interval [28]. With 17‐year follow‐up of Lyon R90‐01 trial, the OS (34% in 6–8 weeks group vs. 34% in 2 weeks group) and local recurrence rates (14.4% vs. 12.1%) were similar between the two groups [29]. The GRECCAR‐6 randomized trial [13] considered two waiting periods of 7 and 11 weeks and showed no difference in the pCR rate, 3‐year OS, disease‐free survival (DFS), and recurrence rates between groups. The long‐term result of RCT by Akgun et al. [30] with a median follow‐up of 80 months revealed that the incidence of DM significantly decreased with nCRT‐surgery interval longer than 8 weeks (30.8% vs. 18.6%), but the LR, 5‐year DFS, or OS were not affected. In our study, 58 (33.1%) deaths occurred during the follow‐up period, which is comparable with the reported mortality rates in previous studies, including those conducted in developed countries [30, 31, 32, 33].

A retrospective study by Luo et al. [9] evaluated the impact of waiting period on oncologic outcomes in poor responders (tumor regression grade 2–3) with LARC. They demonstrated that patients with nCRT‐surgery interval > 8 weeks had significantly inferior OS and DFS compared with patients in the short interval group (≤ 8 weeks). Another cohort study investigated the optimal time interval to achieve the best RFS after nCRT and revealed that the time interval more than 10 weeks was an independent predictor of higher RFS. Patients in the longer interval group had significantly higher 5‐year RFS than those in the short interval group (86.8% vs. 77.8%). But the 5‐year OS was similar between the two groups [20].

Neoadjuvant CRT can shrink rectal tumors to varying extents, making the distance between the tumor's inferior edge and the dentate line more obvious, resulting in a safe DRM [28]. This study indicated that an extended time interval did not improve the anal preservation rate. A meta‐analysis of 13 studies, which enrolled 19 652 patients, found that SPP rate was independent of waiting period [6]. In contrast, Roxburgh et al. [34] indicated that patients with a longer time interval experienced significantly higher APRs. Nevertheless, most previous investigations showed no beneficial effect of an extended waiting period on SPPs [7, 15, 27].

Patients with rectal cancer receiving pelvic radiotherapy may experience pelvic fibrosis or edema, and an adequate interval is needed between the peak effects of radiotherapy and the alleviation of the acute response. If the waiting period is too short, pelvic edema will not fully resolve and may result in a compromised visual field and inadequate exposure of surgical space, hence elevating the risk of perioperative complications. Furthermore, the anal preservation rate may be decreased, and the chance of positive margins and incomplete resection may be raised [28]. Tulchinsky et al. [7] showed the overall morbidity rate of patients with the time interval over 7 weeks decreased (36% vs. 48%), but this did not reach statistical significance. On the other hand, if the waiting period is too long, pelvic tissue fibrosis can develop, and the severity of fibrosis can worsen over time. Severe fibrous scarring can lead to greater difficulty and longer operation time. As well, the likelihood of anastomotic leakage is further increased due to intestinal and rectal wall fibrosis [28]. In the study by Guzmán et al. [4], nCRT‐surgery interval > 12 weeks was associated with an increased risk of minor postoperative complications, incomplete mesorectum, and conversion to open surgery. The phase III GRECCAR6 trial found that delaying surgery until 11 weeks following nCRT was associated with lower specimen quality and increased morbidity and medical complications [13]. Nonetheless, several studies indicated no substantial difference in the occurrence of postoperative complications among patients with different nCRT‐surgery intervals [1, 15, 35, 36]. In line with these reports, the present study found no substantial difference in the R0 resection rate or postoperative complications, including anastomotic leakage, between the two interval groups, suggesting that postponing surgery over 6 weeks was deemed safe and did not result in further complications.

In our study, ypTNM staging was an independent predictor of OS (HR, 7.6 (3–18.7)) and RFS (HR, 3.3(1.2–9)) for ypStage III vs. ypStage 0. Numerous studies indicated that lower pathologic T stage and N stage were correlated with reduced recurrence and enhanced DFS, as well as being an important predictor of survival [3, 37, 38]. Kim et al. [39] demonstrated that 5‐year DFS for patients with stage N0 and *N*+ rectal cancer was 65.2% and 35.7%, respectively (*p* = 0.002). Additionally, ypN stage was an important prognostic factor of survival rate. In a study by Wen et al. [40], pathological stage (ypStage 0, I vs. ypStage II vs. ypStage III) was significantly associated with 5‐year DFS (74.5% vs. 77.4% vs. 50.5%), and 5‐year OS (87.9% vs. 75.5% vs. 56.7%). Another study reported that OS was markedly inferior for ypstage III, with a probability of 64.5%. DFS was also significantly lower in those with ypstage II (65.3%) and ypstage III (54.3%) [41]. Other studies also reported similar findings [42, 43]. One of the biggest challenges with nCRT regimens is the potential for unanticipated interruptions during treatment sessions, which is unfavorable and may adversely affect the therapeutic outcomes. A study by Liscu et al. [41] revealed that LARC patients with tumoral or nodal downstaging had fewer days of treatment interruption compared to those without downstaging. Patients with fewer than 4 days of interruption exhibited a superior OS probability compared to those with 4 or more days (90.2% vs. 57.9%, *p*‐value < 0.001). DFS was significantly higher in LARC patients with interruptions of fewer than 3 days compared to those with interruptions of 3 days or more (82.2% vs. 50.5%).

APR is a well‐known unfavorable prognostic factor of rectal cancer [44], mostly conducted for lower or advanced rectal tumors. Lower rectal cancer exhibits more aggressive behaviors, higher T stages, and a higher likelihood of poorly differentiated histology compared to upper rectal cancer [45]. APR has also been identified as an independent predictor of positive CRM [46]. Accordingly, APR was regarded as an important prognostic factor for RFS in our study. The oncologic outcomes of APR and SPP have been compared in several studies [47, 48]. As reported by Kim et al. [49], patients undergoing APR had a significantly lower 5‐year cancer‐specific survival (52.9% vs. 71.1%) and greater local recurrence (22.0% vs. 11.5%) than those undergoing SPP. Nagtegaal et al. [50] showed the poor prognosis of patients undergoing APR was attributed to higher rates of tissue perforation and positive CRM. Conversely, Chuwa and Seow‐Choen [51] showed that patients who underwent APR had no different oncologic outcomes compared to those with anterior resection. They suggested that using standard surgical techniques in specialized centers, both SPP and APR could be conducted with comparable mortality and morbidity, without affecting oncological prognosis. There is still debate over the oncologic benefits of SPP in comparison to APR.

In these non‐prospective studies, there may have been specific reasons for scheduling surgery at shorter or longer waiting periods. For example, a large, bulky tumor exhibiting partial response during initial evaluation may warrant delaying surgery, while progressive tumors would require early resections [52]. Moreover, rectal tumor locations, radiotherapy doses, sensitizing chemotherapy agents, follow‐up durations, and the cutoff for nCRT‐surgery interval varied throughout different studies. These factors may play a role in the conflicting results. The optimal nCRT‐surgery interval should facilitate maximum tumor regression, with low risk of adverse surgical outcomes and improved long‐term functional and oncological prognosis [30].

Laparoscopic surgery may have technical challenges in patients with large or advanced distal rectal tumors, and pelvic access/exposure is notably limited in obese individuals and in males with a narrow pelvis. Robotic surgery has been specifically designed to address the technical limitations of laparoscopy, providing an advantage particularly in narrow spaces [53]. Laparoscopic female and robotic male sphincter‐preserving surgery for mid‐low rectal cancers were comparable in specimen quality, postoperative complications, and long‐term oncological outcomes [54]. The REAL trial revealed that robotic surgery was significantly associated with lower 3‐year LR (1.6% vs. 4.0%) and higher 3‐year DFS (87.2% vs. 83.4%) compared with laparoscopic surgery. However, there was no substantial difference in 3‐year OS between the robotic and laparoscopic groups (94.7% vs. 93.0%) [55]. In a cohort study by Laks et al. [56], open surgery was associated with higher intra‐ and postoperative complications and longer hospital stay compared with laparoscopic and robotic approaches. No significant difference was observed between groups in positive resection margins, harvested lymph node, 5‐year OS, and 5‐year DFS. They found that male gender was an independent factor for major complications. Gender‐dependent differences in pelvic anatomy play an important role in complication rates, as Akiyoshi et al. [57] demonstrated that pelvic outlet served as an independent predictor of anastomosis leakage. A meta‐analysis conducted by Khajeh et al. [58] revealed that robotic surgery resulted in lower surgical site infection, reduced blood loss, shorter hospitalization, and superior negative resection margins compared to open resection. Additionally, compared to laparoscopic surgery, robotic surgery had higher negative CRM, lower conversion and reoperation rates, and reduced blood loss. However, robotic surgery exhibited longer operation times and higher costs compared to open and laparoscopic procedures. It demands a surgical platform that is not readily available to all colorectal surgical teams.

The main drawback of the current study was its retrospective design, with its potential bias. The number of studied patients was relatively low, and this study may be underpowered to observe the impact of the extended waiting period on survival rates. Large, multicenter studies, including RCTs, are needed to provide a higher level of evidence for determining the optimal time of surgery following nCRT and to improve the clinical management of LARC.

## 5 Conclusions

Our retrospective study showed that pCR achievement rates, OS, and RFS were not affected by nCRT‐surgery interval in LARC patients who underwent laparoscopic surgery in the cohort studied. In addition, time interval of more than 6 weeks may be safe, as no substantial difference in postoperative complications was observed between groups. The ypTNM staging emerged as a statistically significant independent predictor of OS and RFS for ypStage III vs. ypStage 0. Moreover, APR was regarded as a considerable prognostic factor for RFS in our cohort. These findings require further investigation by large‐scale, multicenter, and high‐quality RCTs in the future to determine the optimal time for surgery following nCRT and to improve the clinical management of LARC.

## Author Contributions

**Marzieh Rahimi:** investigation, writing – original draft, writing – review and editing; **Mohammadreza Mamaghani‐Ghazijahani:** validation, investigation, data curation; **Fatemeh Shahabi:** methodology, software, formal analysis; **Majid Ansari:** conceptualization, writing – review and editing; **Mahdie Ghiyasi Noei:** data curation, investigation; **Mina Alvandipour:** supervision, validation; **Abbas Abdollahi:** conceptualization, supervision, resources, project administration.

## Funding

The authors have nothing to report.

## Ethics Statement

This study was approved by the Ethics Committee of Mashhad University of Medical Sciences (IR.MUMS.IRH.REC.1403.061) and conducted in accordance with the Declaration of Helsinki. The informed consent was waived by the Ethics Committee of Mashhad University of Medical Sciences due to the observational and retrospective nature of the study. In accordance with relevant guidelines and regulations, identifiable data, including patient personal information, was removed from the dataset and replaced with a unique code for each patient. Only the corresponding author (A.A.) and another author (F.Sh.) who followed up the patients had access to personal data, and the others were blinded to this information.

## Conflicts of Interest

The authors declare no conflicts of interest.

## Data Availability

The data that support the findings of this study are available from the corresponding author upon reasonable request.
